# Thrombectomy in a severe case of iliofemoral venous thrombosis involving the deep femoral vein via a single percutaneous access from the jugular vein: case report and description of the technique

**DOI:** 10.1590/1677-5449.202101921

**Published:** 2022-05-06

**Authors:** Fabio Henrique Rossi

**Affiliations:** 1 Instituto Dante Pazzanese de Cardiologia – IDPC, São Paulo, SP, Brasil.; 2 Instituto de Excelência em Doenças Venosas ­– IEDV, São Paulo, SP, Brasil.

**Keywords:** angioplasty, thrombectomy, iliac vein, thrombosis

## Abstract

Iliac vein thrombectomy is usually performed via access through veins located in the lower limbs, which makes it impossible to treat the deep femoral vein, which in turn is an important inflow route to the iliac vein stent. We describe a clinical case and the previously unpublished technique of percutaneous thrombectomy, angioplasty, and stent implantation performed in a single session and with a single access, obtained via the internal jugular vein.

## INTRODUCTION

Mechanical thrombectomy can be used to treat iliofemoral deep venous thrombosis (DVT) in a single session.^1^^-^3 Access is conventionally obtained via veins located in the lower limbs, primarily the popliteal vein, preventing access to the ipsilateral deep femoral vein, which, in turn, is consistently involved in severe cases. Although contralateral puncture enables access to the deep femoral vein, it prevents angioplasty and stent deployment in the great majority of cases, when there is an obstruction in the iliocaval segment. We describe a clinical case treated using a technique comprising percutaneous thrombectomy, angioplasty, and stenting performed in a single session and via a single access, obtained via the internal jugular vein.

## CLINICAL CASE

A 16-year-old female student presented complaining of pain and edema of the left lower limb with onset 1 day before. She stated that she had no history of similar occurrences or of previous surgery, but reported that she had put on weight and had been taking oral contraception for the last 4 months. Physical examination revealed considerable edema of the thigh and left leg and reversible cyanosis of the toes. All pulses were present. Vascular echography showed a large deep vein thrombosis (DVT) involving the popliteal, femoral, and iliac veins of the left leg. She was admitted and given full anticoagulation with low molecular weight heparin and maintained at rest, in the Trendelenburg position. After 48 hours in hospital, her clinical status was unchanged and she had difficulty walking, even in the hospital room.

## DESCRIPTION OF THE TECHNIQUE

**Figure 1:**
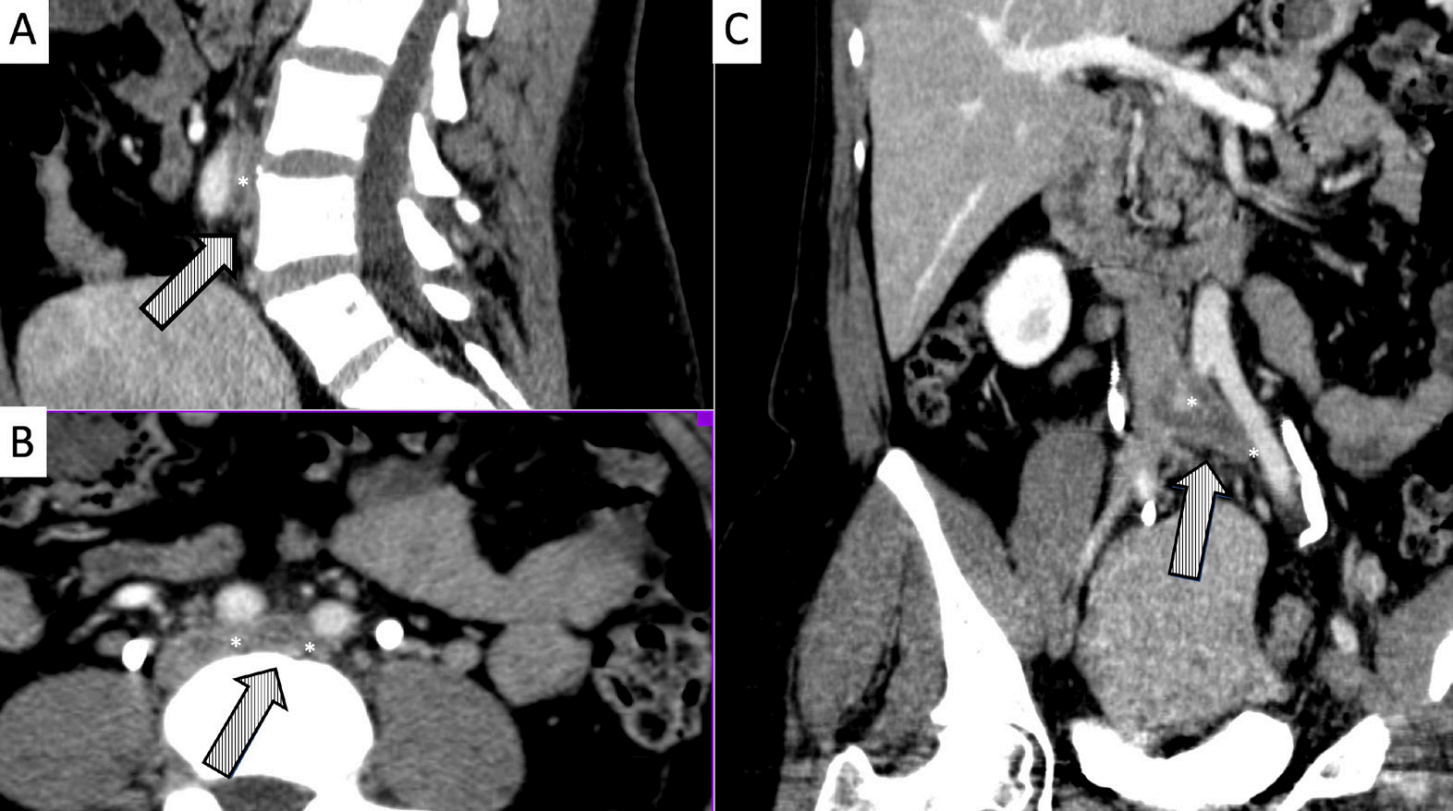
Abdominal and pelvic angiotomography showing presence and size of the thrombosis and angiotomographic classification at the point of greatest compression (*) in the cavo-ilio-femoral segment. **A)** Coronal axis; **B)** Sagittal axis; **C)** Transverse axis.

**Figure 2:**
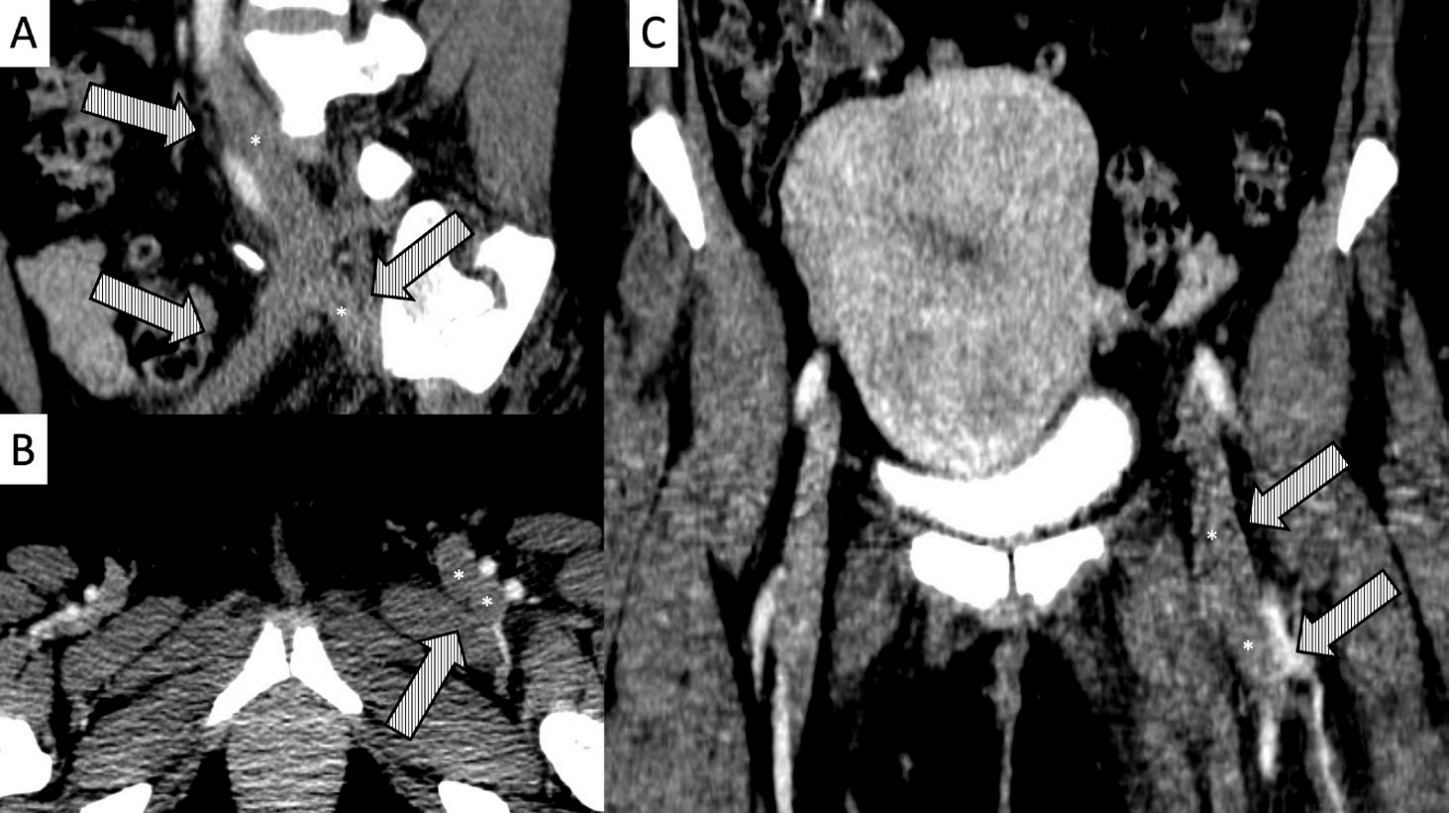
Angiotomography demonstrating presence of thrombus filling the entire lumen of the left common femoral, femoral, and deep femoral vein (*). **A)** Coronal axis; **B)** Sagittal axis; **C)** Transverse axis.

**Figure 3:**
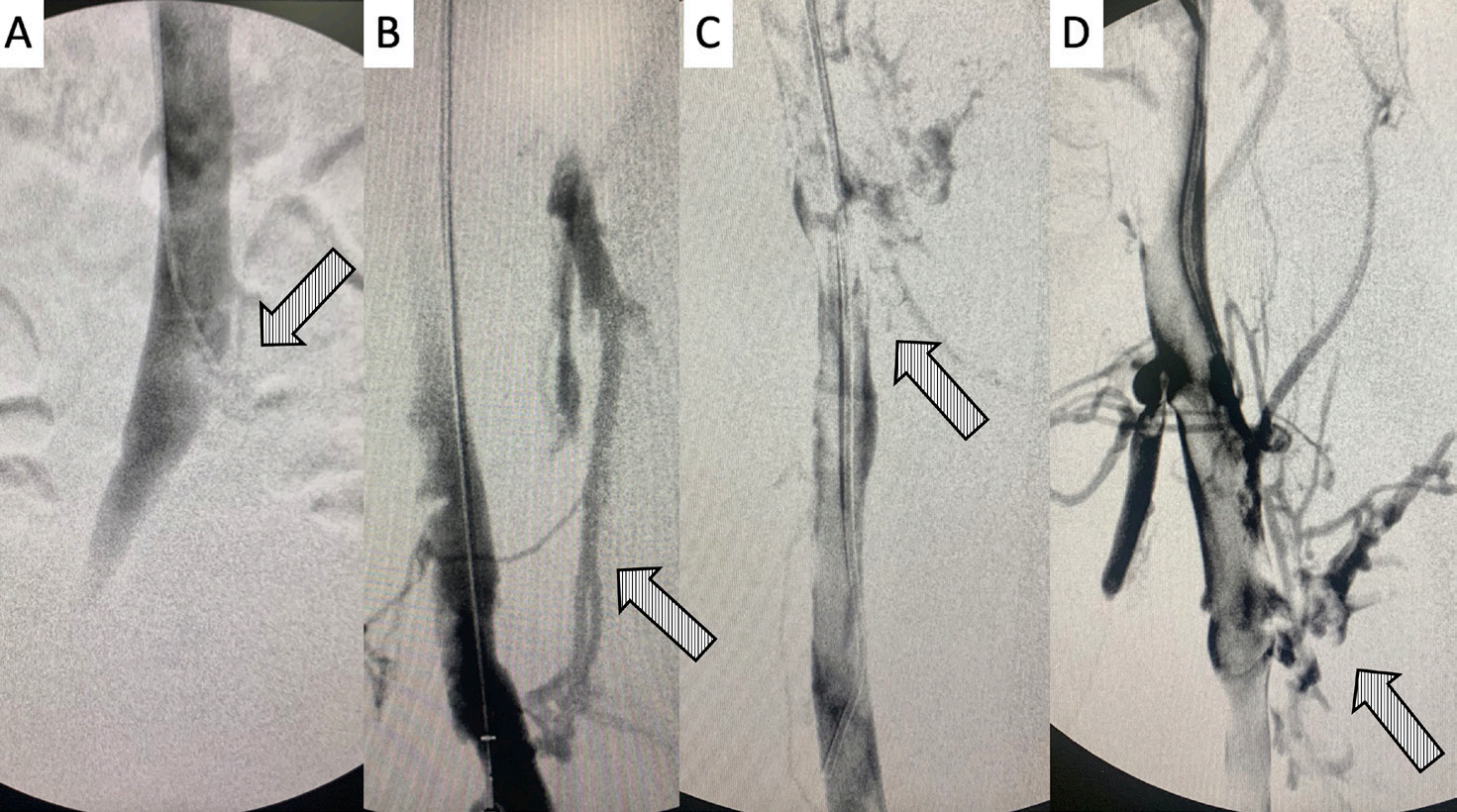
Digital subtraction venography. **A)** Tip of the 5F PM catheter positioned at the confluence of the common iliac veins, showing the site of compression by the right iliac artery; **B)** initial venography conducted with the Angiojet Zelante thrombectomy catheter, positioned in the left popliteal vein; note the presence of collateral flow between this vein and the deep femoral vein, which is obstructed by thrombi; **C** and **D)** presence of a large volume of thrombi in the left femoral and deep femoral veins.

**Figure 4:**
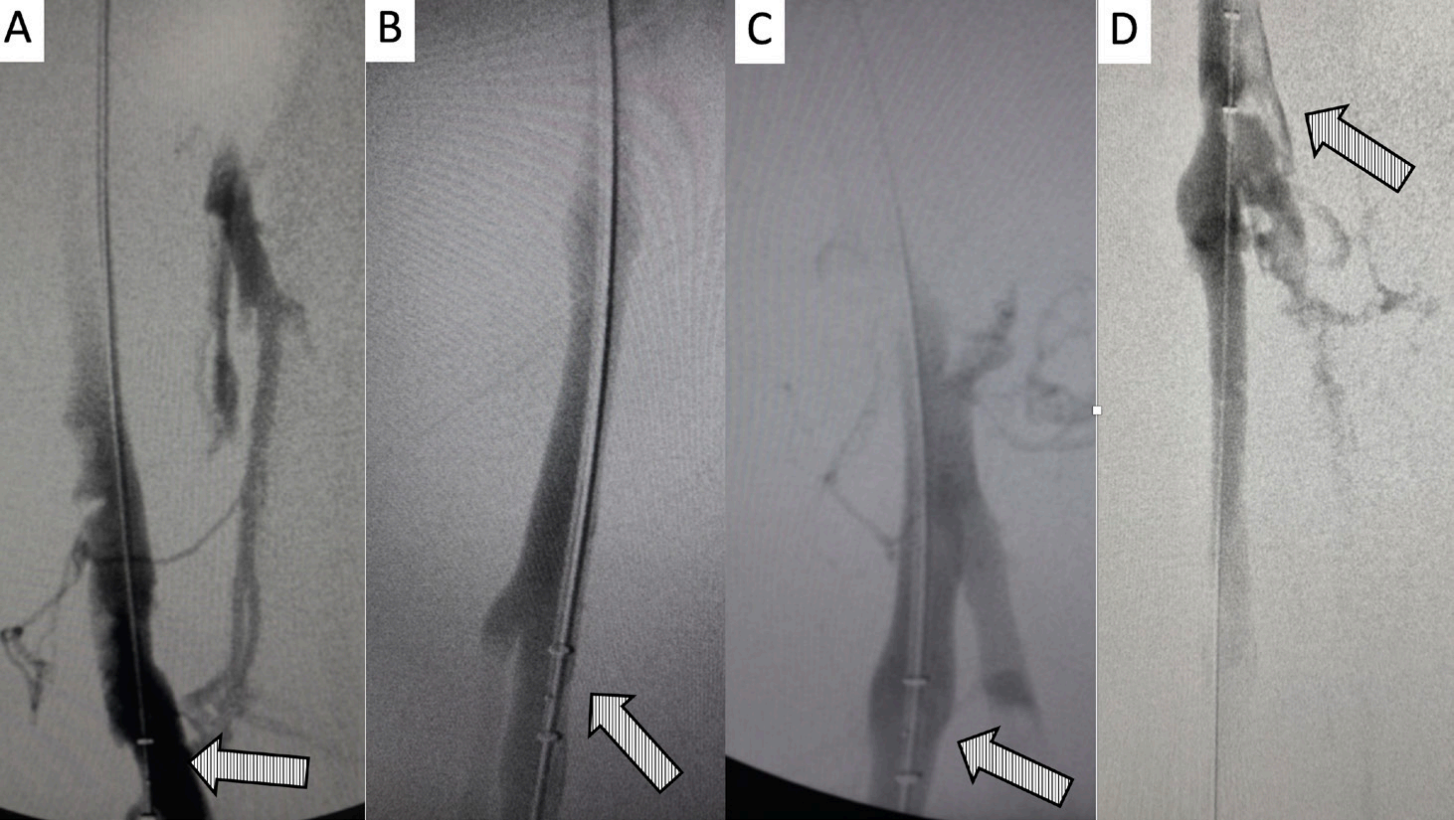
Digital subtraction venography demonstrating **A)** initial presence of obstruction of left femoral and deep femoral veins by thrombi; in **B, C** and **D)** intraoperative control venography after infusion of the thrombolytic agent with the “pulse spray” technique and mechanical aspiration of the left femoropopliteal segment.

**Figure 5:**
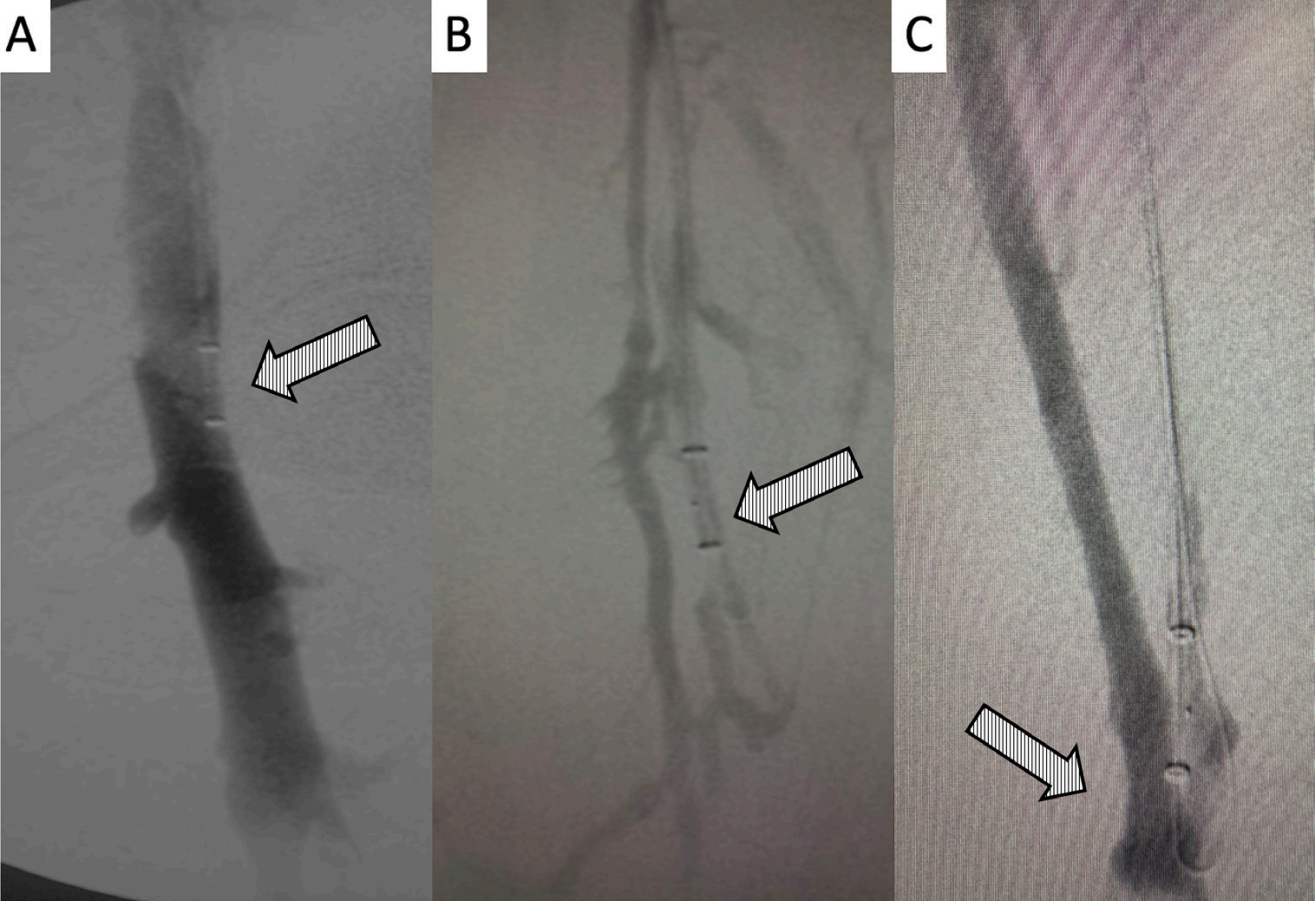
Digital subtraction venography demonstrating the technique for repositioning the Angiojet catheter tip in the caudal extremity of the left deep common femoral vein: **A)** catheter tip placed in the left common femoral vein without the guidewire; **B** and **C)** positioning of the tip of the same catheter over the hydrophilic guidewire in the caudal extremity of the left deep femoral vein.

**Figure 6:**
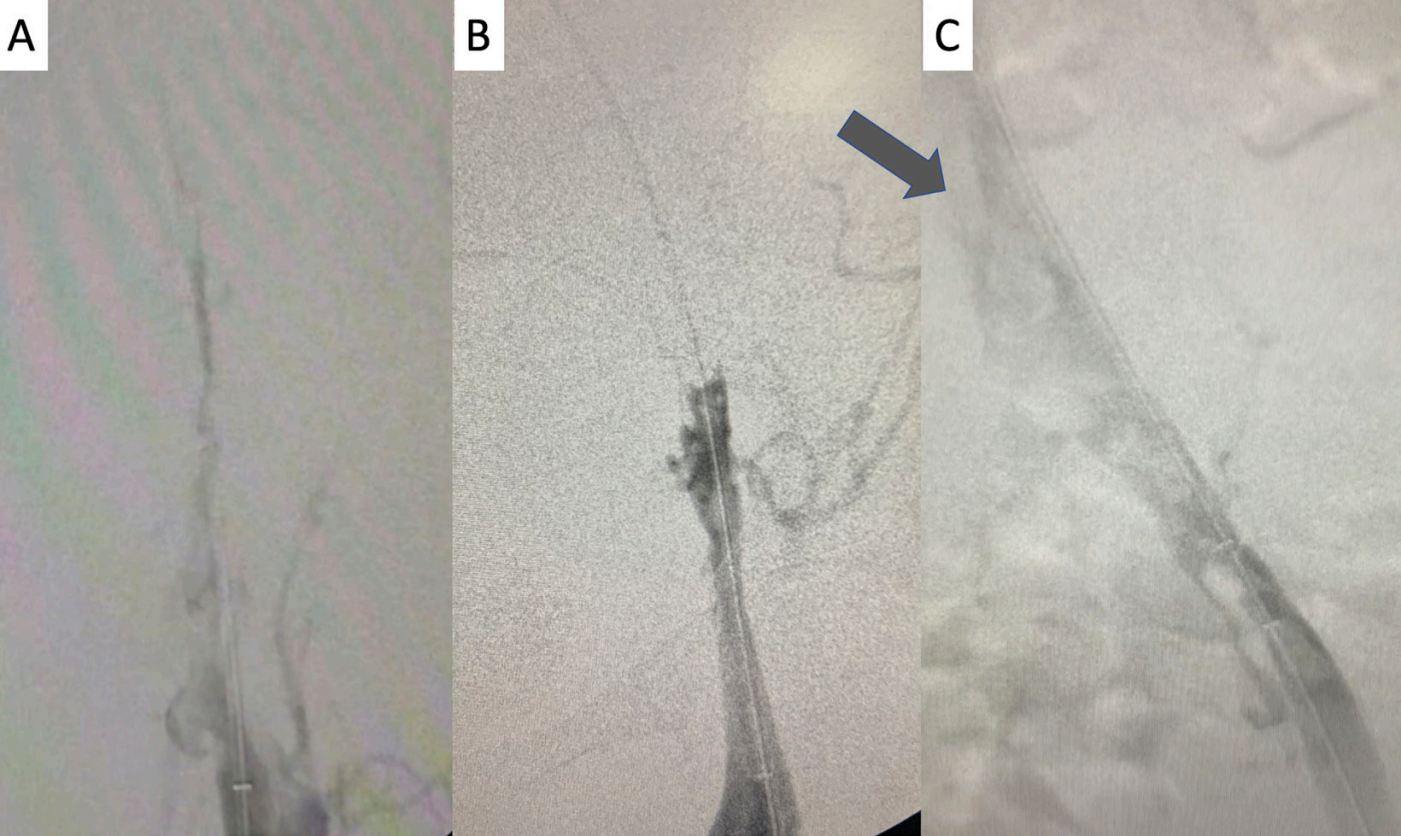
Digital subtraction venography of the cavo-ilio-femoral axis: **A** and **B)** initial venography demonstrating occlusion of the iliofemoral axis; **C)** venographic appearance after infusion of thrombolytic with the “pulse spray” technique and mechanical aspiration. At the arrow, we can observe the anatomic obstruction that occurs at the “mouth” of the left common iliac vein, provoking compression by the right common iliac artery and used as the cranial limit of advancement of the thrombectomy catheter, with the objective of preventing fragments of thrombus entering the inferior vena cava.

**Figure 7:**
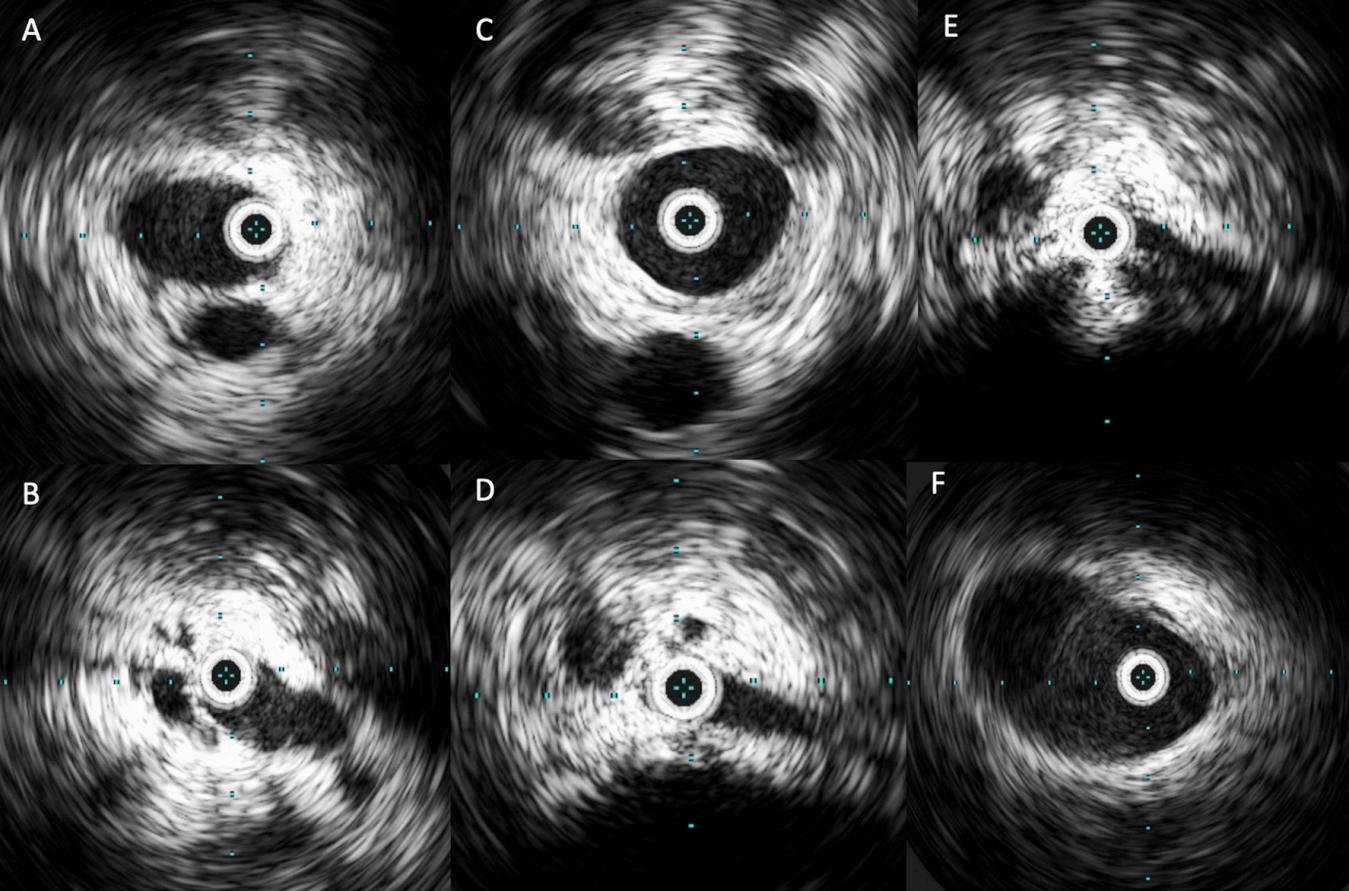
Image acquired when crossing the entire treated segment with the intravascular ultrasonography catheter (Vision PV.035 – Philips) with the objective of identifying remnant thrombi and residual points of compression and/or obstruction: **A)** left femoral vein; **B)** left common femoral vein below the inguinal ligament; **C)** mid third of the left common iliac vein; **D** and **E)** severe compression and obstruction (>80%) at the confluence of the iliac veins and the caudal extremity of the inferior vena cava; **D)** inferior vena cava in mid infrarenal third.

**Figure 8:**
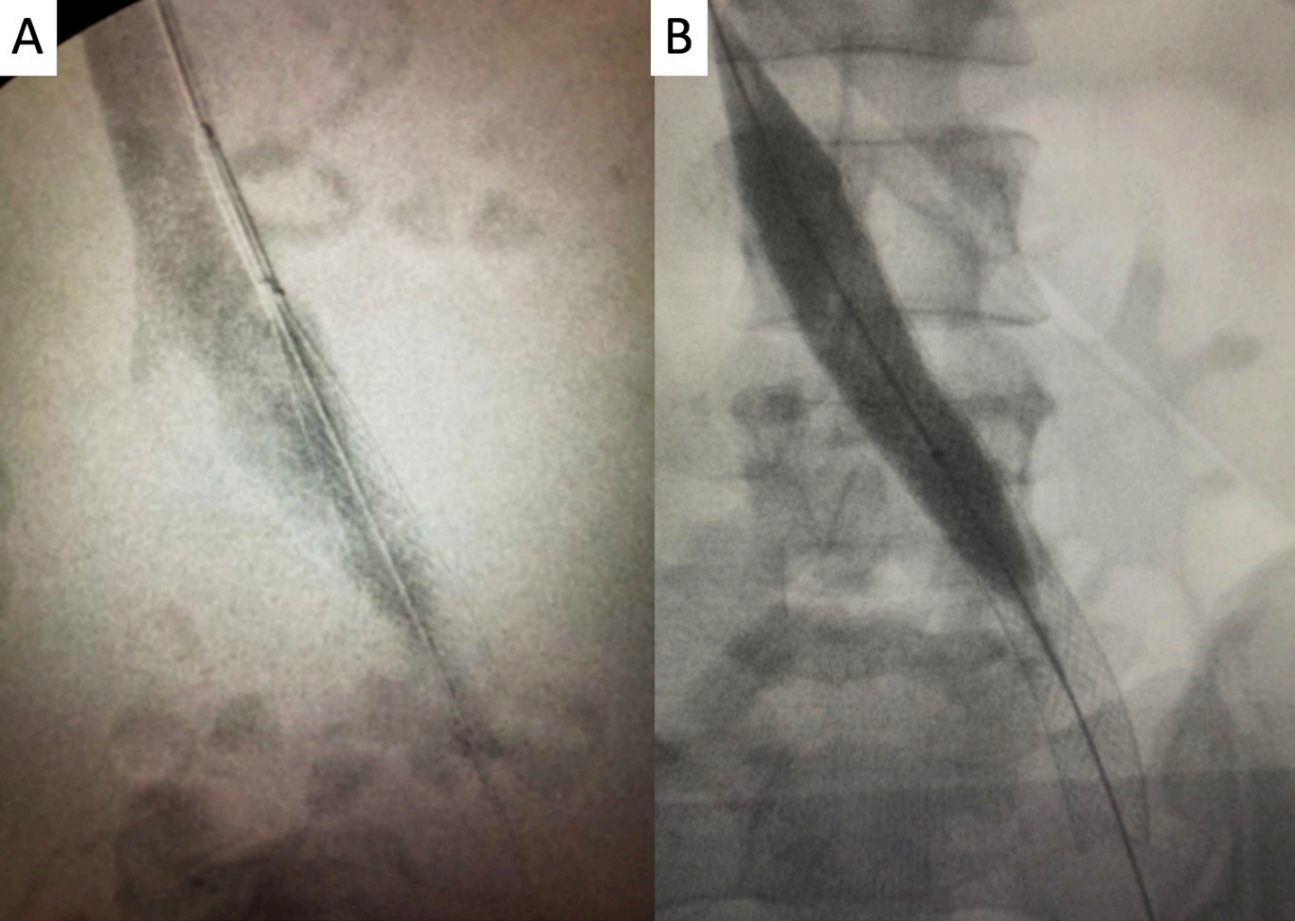
**A)** Digital subtraction venography demonstrating controlled-release of the Wallstent 18 x 90 mm; and **B)** post-ballooning of the stent with an 18 x 40 mm XXL catheter balloon.

**Figure 9:**
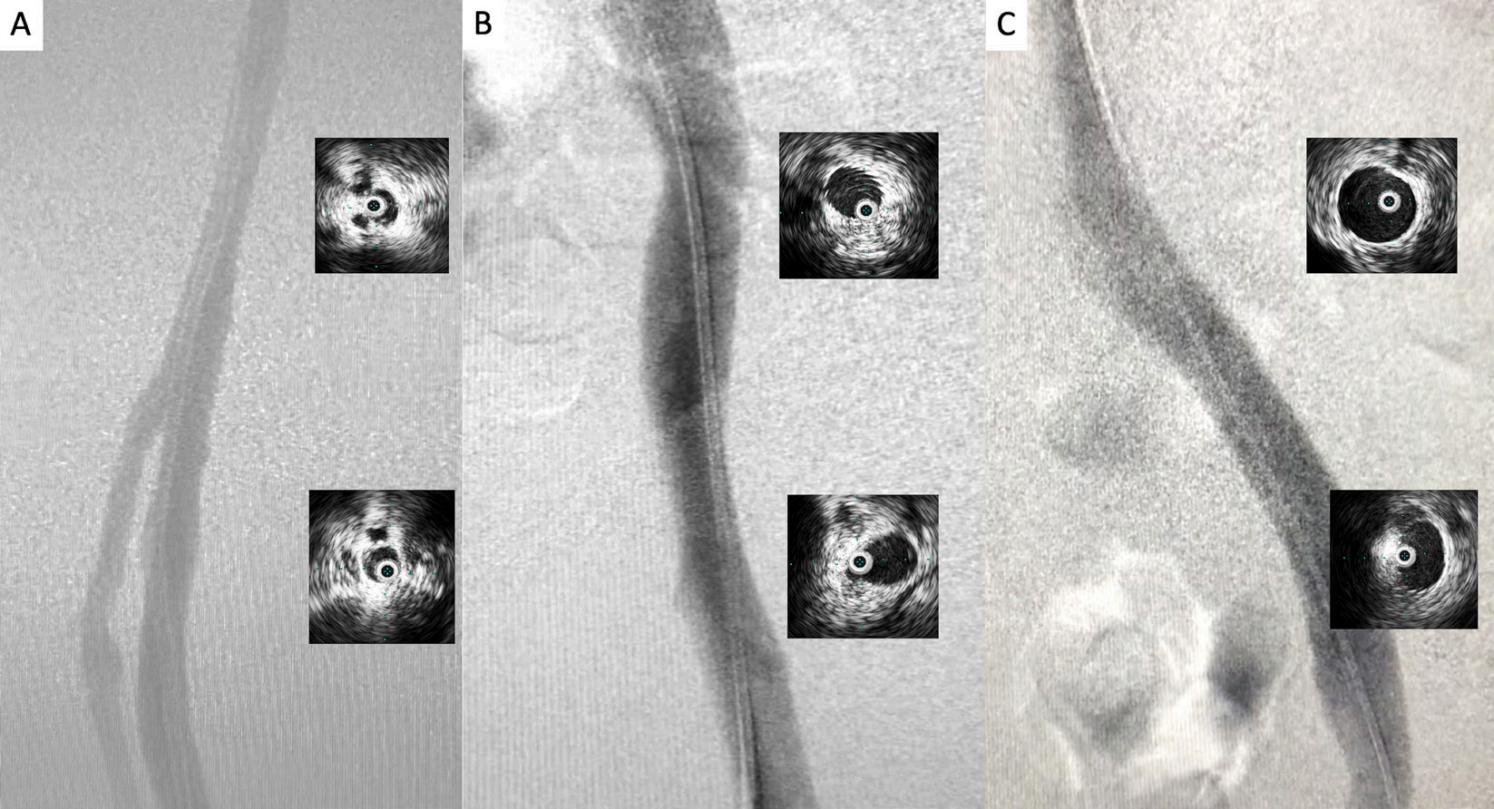
Digital subtraction venography and final intravascular ultrasonography control along the femoral **(A)**, iliofemoral **(B)**, and vena cava segments **(C)**, demonstrating adequate flow and recovery of the cross-sectional area of the segments treated.

Angiotomography was used to identify and classify^4^^,^^5^ the obstructed venous segments (Figures 1 and 2). The technique employed comprised the following steps: 1) ultrasound-guided puncture of the right internal jugular vein (RIJV), systemic heparinization and selective catheterization of the popliteal vein (11F x 11 cm introducer, 5F x 110 cm multipurpose catheter, 0.035” x 260 cm Terumo stiff hydrophilic guidewire (Terumo Medical, Tokyo, Japan), and 0.035” x 260 cm super stiff Amplatz guidewire (Boston Scientific, Marlborough, USA), positioning of an Angiojet Zelante catheter (Boston Scientific, Marlborough, United States) over the Amplatz guidewire and retrograde venography via the catheter, with identification of the obstructed segment (Figure 3); 2) preparation of the Alteplase 10 mg solution in 100 mL of saline 0.9%, infusion of 20 mL of this solution using the “pulse spray” technique into the thrombus interior up to the common femoral vein, a 20-minute wait, aspiration of the thrombus via the Angiojet catheter (Boston Scientific, Marlborough, USA), confirmation of the result by venography performed with the same catheter (Figure 4); 3) repositioning of the catheter tip in the common femoral vein, substitution of the Amplatz guidewire with the stiff hydrophilic wire and positioning of the tip of the stiff guidewire, repositioning of the Angiojet catheter at the caudal extremity of the deep femoral vein and repetition of step 2 in this segment (Figure 5); 4) venography performed with the Angiojet catheter placed in the common femoral vein, observing the characteristics of the obstruction in this iliocaval segment, repetition of step 2, taking the site of greatest compression observed on angiotomography as the cranial limit, without yet penetrating the lumen of the inferior vena cava to avoid release of remnants of the macerated thrombus into the bloodstream (Figure 6); 5) crossing the entire treated segment with the intravascular ultrasonography (IVUS) catheter (Vision PV.035 – Philips, Holland), with the objective of identifying residual thrombi and points of compression and/or obstruction (Figure 7); 6) angioplasty with a balloon catheter (XXL, 14 to 20 x 40 mm – Boston Scientific or Atlas Gold 14 to 20 x 40 mm – BD; Mustang 8 to 12 x 60mm – Boston Scientific) of obstructed segments identified with IVUS; 7) deployment of a 14 to 20 x 90 mm self-expanding stent (Wallstent – Boston Scientific; Venovo – BD; Zilver Vena – Cook; Abre – Medtronic) in the obstructed iliocaval segment; 8) post-ballooning of the stent with the same balloon catheter (Figure 8); 9) final passage of the IVUS catheter and final control venography (Figure 9); and 10) closure of the puncture site with a Perclose vascular closure device (Abbott Medical, MS, USA).

## POSTOPERATIVE COURSE

**Figure 10:**
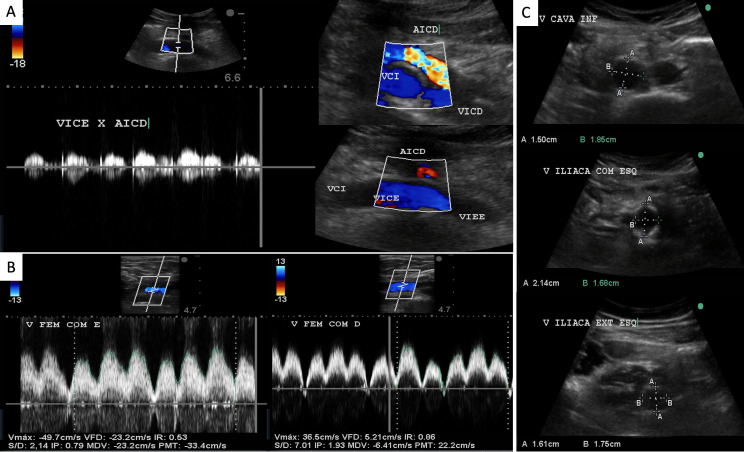
Postoperative control Doppler ultrasonography demonstrating **(A)** patency of the inferior vena cava (VCI), the stent venous positioned in the left common iliac vein (VICE), from its cranial segment over the right common iliac artery (AICD), and in the left external iliac vein (VIEE), and the right common iliac vein (VICD); **B)** Venous flow in phase with respiratory movements in the left (VFCE) and right (VFCD) common femoral veins; **C)** diameters of the stent in the VCI, VICE, and VIEE.

During the immediate postoperative period, the patient’s symptoms and edema improved and she was discharged from hospital on the second day after the procedure. Recently, one and a half years after hospital discharge, she remained asymptomatic and control vascular echography showed total recanalization of the treated segment, stent patency, and no points of obstruction (Figure 10). This innovative technique was developed as part of a research project on diagnosis and treatment of iliac venous obstructions, approved by the Ethics Committee (4101/2012), and financed by the Fundação de Amparo à Pesquisa do Estado de São Paulo (FAPESP) (2012/01021-9).

## DISCUSSION

Invasive and percutaneous treatment for iliofemoral DVT is becoming increasingly popular for severe symptomatic patients, defined as those needing to be admitted for pain, edema, cyanosis, and difficulty walking and whose symptoms do not improve despite full anticoagulation and limb elevation. In this situation, there is a high risk of occurrence of severe postthrombotic syndrome, with considerable loss of quality of life, relapse of thrombosis, and high economic and social costs.^2^ The development of mechanical thrombectomy devices and improvements to the technique have enabled treatment to be completed in a single session, using minimal quantities of thrombolytics, delivering shorter procedure times, shorter hospital stays, and lower treatment costs.^1^^,^^3^^,^^6^ Thrombosis of the deep femoral vein is linked to the severity of symptoms in both the acute and chronic phases, with a higher incidence of postthrombotic syndrome, and with worse treatment patency outcomes after angioplasty and placement of the stent in the iliofemoral axis.^1^^,^^7^

In the standard technique used for percutaneous treatment, access is conventionally obtained by puncture of veins located in the lower limbs, primarily the popliteal vein, which prevents access to the ipsilateral deep femoral vein. Although contralateral puncture does allow access to this vein, it does not permit angioplasty and stent deployment when there is obstruction of the iliocaval axis, which is present in the great majority of cases.

The hitherto unpublished technique described here enables use of a single access, obtained via the internal jugular vein, and treatment in a single session of the entire iliofemoral axis, including the whole length of the deep femoral vein. The characteristics of the Angiojet device enable perioperative venographic control, thrombolysis with the “pulse spray” technique, and mechanical aspiration to be performed with the same catheter and access. Early extraction of the thrombus from the common and deep femoral veins is very important, because it immediately restores adequate blood flow to the iliac segment subjected to angioplasty and stenting, ensuring its short and long term patency. Use of IVUS is very important for success of the technique, because we know that venography can fail to identify points of compression and residual thrombi, and only this method is capable of identifying possible residual obstructions. Another important factor that should be considered is that presence of the thrombus in the vessel lumen can trigger an inflammatory process in its walls within the first hours, provoking phlebosclerosis and elastic recoil, in a process first recognized by Rokitansky,^8^ which can make angioplasty difficult because of intense fibrotic retraction when performed in the chronic phase.

## CONCLUSIONS

Mechanical thrombectomy of iliac DVT performed using the single-session, single-puncture technique enables immediate treatment of the deep femoral vein, improving the patient’s clinical status, guaranteeing adequate flux and possibly improving short and long term stent patency outcomes.
