# Supplementary material for: Thrombectomy in a severe case of iliofemoral venous thrombosis involving the deep femoral vein via a single percutaneous access from the jugular vein: case report and description of the technique
Source: J Vasc Bras. 2022 May 6;21:e20210192. doi: 10.1590/1677-5449.202101921 (PMC9083802; doi:10.1590/1677-5449.202101921)
Supplement: Translation

# Translation: Trombectomia em caso grave de trombose venosa iliofemoral afetando a veia femoral profunda pela técnica de punção e sessão única realizada pela veia jugular: relato de caso e descrição da técnica

**DOI:** 10.1590/1677-5449.202101922

**Authors:** Fabio Henrique Rossi

**Affiliations:** 1 Instituto Dante Pazzanese de Cardiologia – IDPC, São Paulo, SP, Brasil.; 2 Instituto de Excelência em Doenças Venosas ­– IEDV, São Paulo, SP, Brasil.

**Keywords:** angioplastia, trombectomia, veia ilíaca, trombose

## Abstract

A trombectomia mecânica venosa ilíaca geralmente é realizada por acesso em veias localizadas nos membros inferiores, o que impossibilita o tratamento da veia femoral profunda, que, por sua vez, é uma importante via de influxo ao stent venoso ilíaco. Descrevemos um caso clínico em que foi aplicada a técnica inédita de trombectomia percutânea, angioplastia e implante de stent, realizada por sessão e acesso único, obtido na veia jugular interna.

## INTRODUÇÃO

A trombectomia mecânica permite o tratamento da trombose venosa profunda (TVP) ilíaco-femoral em uma única sessão^1^^-^3. O acesso convencionalmente é obtido em veias localizadas nos membros inferiores, principalmente na veia poplítea, o que impede o acesso à veia femoral profunda ipsilateral, que, por sua vez, é constantemente envolvida nos casos graves. A punção contralateral, apesar de possibilitar o acesso a essa veia, impossibilita a angioplastia e a liberação do stent na grande maioria dos casos, quando existe obstrução no eixo ilíaco-cavo. Descrevemos um caso clínico em que foi aplicada a técnica de trombectomia percutânea, angioplastia e implante de stent, realizada por sessão e acesso único, obtido na veia jugular interna.

## CASO CLÍNICO

Uma paciente de 16 anos, do sexo feminino e estudante apresentou como queixas dor e edema em membro inferior esquerdo há 1 dia. Negou história clínica semelhante ou cirurgia prévia e relatou que havia ganhado peso e vinha fazendo uso de anticoncepcional oral nos últimos 4 meses. Ao exame físico, apresentava edema volumoso em coxa e perna esquerda e cianose não fixa de pododáctilos. Todos os pulsos eram presentes. A ecografia vascular revelou extensa TVP envolvendo as veias da perna, poplítea, femorais e ilíacas esquerda. A paciente foi internada, submetida a anticoagulação plena com heparina de baixo peso molecular e mantida em repouso, em posição de Trendelenburg. Após 48 horas de internação, a paciente manteve o quadro clínico e havia restrição para a locomoção, mesmo dentro do quarto.

## DESCRIÇÃO DA TÉCNICA

Foram realizadas a identificação e a classificação angiotomográfica^4^^,^^5^ (Figura 1 e 2) do segmento venoso obstruído. A técnica empregada seguiu estas etapas: 1) punção ecoguiada da veia jugular interna direita (VJID), heparinização sistêmica e cateterização seletiva da veia poplítea (introdutor 11F x 11 cm, cateter MP 5F x 110 cm, guia hidrofílico Terumo *stiff* (Terumo Medical, Tóquio, Japão) de 0,035” x 260 cm e Amplatz *super stiff* 0,035” x 260 cm (Boston Scientific, Marlborough, EUA), posicionamento de cateter Angiojet Zelante (Boston Scientific, Marlborough, EUA) sobre o fio guia Amplatz, venografia retrógrada pelo mesmo, com identificação do segmento obstruído (Figura 3); 2) preparo de solução de Alteplase 10 mg em 100 mL de soro fisiológico a 0,9%, infusão de 20 mL dessa solução pela técnica “*pulse spray*” no interior do trombo até a veia femoral comum, espera de 20 minutos, aspiração do trombo pelo cateter Angiojet (Boston Scientific, Marlborough, EUA), verificação do resultado por venografia realizada pelo próprio cateter (Figura 4); 3) reposicionamento de sua ponta na veia femoral comum, substituição do guia Amplatz pelo hidrofílico *stiff* e posicionamento da ponta desse guia, substituição do cateter Angiojet na extremidade caudal da veia femoral profunda e repetição da etapa 2 nesse segmento (Figura 5); 4) venografia realizada pelo cateter Angiojet estacionado na veia femoral comum, observando as características da obstrução no segmento ilíaco-cavo, repetição da etapa 2, tendo por limite cranial o local de maior compressão observado na angiotomografia, sem nesse momento penetrar a luz da veia cava inferior para que não haja liberação dos resquícios do trombo macerado na corrente sanguínea (Figura 6); 5) passagem de cateter de ultrassonografia intravascular (IVUS) (Vision PV.035 – Philips, Holanda) em todo segmento tratado, com o objetivo de identificar trombos residuais e pontos de compressão e/ou obstrução (Figura 7); 6) angioplastia com cateter balão (XXL, 14 a 20 x 40 mm – Boston Scientific ou Atlas Gold 14 a 20 x 40 mm – BD; Mustang 8 a 12 x 60mm – Boston Scientific) dos segmentos obstrutivos identificados pelo IVUS; 7) implante de stent autoexpansível 14 a 20 x 90 mm (Wallstent – Boston Scientific; Venovo – BD; Zilver Vena – Cook; Abre – Medtronic) no segmento cavo-ilíaco obstruído; 8) pós-balonamento do stent com o mesmo cateter balão (Figura 8); 9) passagem final do cateter de IVUS e venografia final de controle (Figura 9); e 10) fechamento de sítio de punção com oclusor vascular tipo Perclose (Abbott Medical, MN, EUA).

**Figura 1:** Angiotomografia abdomino-pélvica demonstrando presença e extensão da trombose e classificação angiotomográfica de ponto de maior compressão (*) no segmento cavo-ilíaco-femoral. **A)** Eixo coronal; **B)** eixo sagital; **C)** eixo transverso.

**Figura 2:** Angiotomografia demonstrando presença de trombo preenchendo toda a luz da veia femoral, comum, femoral e profunda esquerda (*). **A)** Eixo coronal; **B)** eixo sagital; **C)** eixo transverso.

**Figura 3:** Venografia por subtração digital. **A)** Ponta de cateter MP 5F posicionado em confluência de veias ilíacas comuns, demonstrando o local de compressão pela artéria ilíaca direita; **B)** venografia inicial realizada pelo próprio cateter de trombectomia Angiojet Zelante, posicionado em veia poplítea esquerda; notar a presença de colateral entre essa veia e a veia femoral profunda que se encontra obstruída por trombos; **C e D)** presença de grande volume de trombos em veia femoral e femoral profunda esquerda.

**Figura 4:** Venografia por subtração digital demonstrando **A)** presença inicial de obstrução de veia femoral e femoral profunda esquerda por trombos; em **B, C** e **D)** venografia de controle intraoperatória após realização de infusão de agente trombolítico pela técnica “*pulse spray*” e aspiração mecânica no segmento femoropoplíteo esquerdo.

**Figura 5:** Venografia por subtração digital demonstrando a técnica de reposicionamento da ponta do cateter Angiojet em extremidade caudal da veia femoral comum profunda esquerda: **A)** ponta do cateter estacionada em veia femoral comum esquerda sem o fio guia; **B** e **C)** posicionamento da ponta do mesmo cateter sobre guia hidrofílica em extremidade caudal de veia femoral profunda esquerda.

**Figura 6:** Venografia por subtração digital de eixo cavo-ilíaco-femoral: **A** e **B)** venografia inicial demonstrando oclusão em eixo ilíaco-femoral; **C)** aspecto venográfico após infusão de trombolítico pela técnica “*pulse spray*” e trombectomia mecânica. Na seta, podemos notar a obstrução anatômica que ocorre na “boca” da veia ilíaca comum esquerda, provocada pela compressão da mesma pela artéria ilíaca comum direita, usada como limite cranial da passagem do cateter de trombectomia, com objetivo de evitar que fragmentos de trombo invadam a veia cava inferior.

**Figura 7:** Imagem obtida durante a passagem do cateter de ultrassonografia intravascular (Vision PV.035 – Philips) em todo segmento tratado, com o objetivo de identificar trombos residuais e pontos residuais de compressão e/ou obstrução: **A)** veia femoral esquerda; **B)** veia femoral comum esquerda abaixo do ligamento inguinal; **C)** terço médio da veia ilíaca comum esquerda; **D** e **E)** compressão e obstrução severa (>80%) em confluência de veias ilíacas e em extremidade caudal da veia cava inferior; **D)** veia cava inferior em terço médio infrarrenal.

**Figura 8:** **A)** Venografia por subtração digital demonstrando liberação controlada de Wallstent 18 x 90 mm; e **B)** pós-balonamento do stent com cateter balão XXL 18 x 40 mm.

**Figura 9:** Venografia por subtração digital e passagem de cateter de ultrassonografia intravascular de controle final em segmento femoral **(A)**, ilíaco-femoral **(B)** e cavo **(C)**, demonstrando fluxo adequado e recuperação da área de secção transversa dos segmentos tratados.

## EVOLUÇÃO PÓS-OPERATÓRIA

No pós-operatório imediato, a paciente evoluiu com melhora dos sintomas e do edema e obteve alta hospitalar no segundo dia após a realização do procedimento. Recentemente, após 1 ano e meio da alta hospitalar, mantém-se assintomática, e a ecografia vascular de controle revelou recanalização total do segmento tratado, perviedade do stent e ausência de pontos de obstrução (Figura 10). Essa inovação técnica foi desenvolvida como parte de um projeto de pesquisa sobre o diagnóstico e tratamento de obstruções venosas ilíacas, aprovado pelo Comitê de Ética (4101/2012) e financiado pela Fundação de Amparo à Pesquisa do Estado de São Paulo (FAPESP) (2012/01021-9).

**Figura 10:** Eco-Doppler venoso de controle pós-operatório demonstrando **(A)** perviedade de veia cava inferior (VCI), em stent venoso posicionado em veia ilíaca comum esquerda (VICE), desde seu segmento cranial sob a artéria ilíaca comum direita (AICD), e em veia ilíaca externa esquerda (VIEE), bem como em veia ilíaca comum direita (VICD); **B)** fluxo venoso fásico aos movimentos respiratórios em veia femoral comum esquerda (VFCE) e direita (VFCD); **C)** diâmetros do stent em VCI, VICE e VIEE.

## DISCUSSÃO

O tratamento invasivo e percutâneo da TVP ilíaco-femoral vem ganhando popularidade em pacientes sintomáticos graves, considerados aqueles que necessitam de internação por dor, edema, cianose, dificuldade de deambulação e que não melhoram dos sintomas apesar da anticoagulação plena e elevação do membro. Nessa situação, existe alto risco de que ocorra síndrome pós-trombótica grave, com perda considerável da qualidade de vida, recidiva de trombose e alto custo econômico e social^2^. O desenvolvimento de dispositivos de trombectomia mecânica e o aprimoramento da técnica vêm permitindo que o tratamento seja feito em uma única sessão, com o uso de quantidades mínimas de agentes trombolíticos, permitindo menor tempo de procedimento e de internação e menor custo de tratamento^1^^,^^3^^,^^6^. A trombose da veia femoral profunda está relacionada com a gravidade dos sintomas nas fases aguda e crônica, com uma maior incidência de síndrome pós-trombótica e com piores resultados de perviedade do tratamento após a angioplastia e implante de stent no eixo ilíaco-femoral^1^^,^^7^.

Na técnica usual de tratamento percutâneo, o acesso é convencionalmente obtido pela punção de veias localizadas nos membros inferiores, principalmente da veia poplítea, que impossibilita o acesso a veia femoral profunda ipsilateral. A punção contralateral, apesar de possibilitar o acesso a essa veia, não permite a angioplastia e a liberação do stent quando existe obstrução no eixo ilíaco-cavo, presente na grande maioria dos casos.

A inédita técnica aqui descrita permite o acesso único, obtido na veia jugular interna, e o tratamento em uma única sessão de todo o eixo ilíaco-femoral, incluindo a veia femoral profunda em toda a sua extensão. As características do dispositivo Angiojet permitem que o controle venográfico perioperatório, a trombólise pela técnica “*pulse spray*” e a aspiração mecânica sejam feitos pelo mesmo cateter e via de acesso. A extração precoce do trombo na veia femoral comum e profunda é muito importante, pois permite afluxo sanguíneo adequado e imediato ao segmento ilíaco submetido a angioplastia e implante de stent, assegurando sua perviedade a curto e longo prazo. O uso do IVUS é muito importante para o sucesso da técnica, pois sabemos que a venografia pode falhar na identificação dos pontos de compressão e trombos residuais, e apenas esse método é capaz de identificar possíveis obstruções residuais. Um outro fator importante que deve ser considerado é que a permanência de trombo na luz do vaso pode provocar processo inflamatório em sua parede já nas primeiras horas, podendo provocar flebosclerose e recolhimento elástico, processo primeiramente reconhecido por Rokitansky^8^, que pode dificultar a angioplastia pela presença de intensa retração fibrótica quando realizada na fase crônica.

## CONCLUSÃO

A técnica de trombectomia mecânica da TVP ilíaca realizada pela técnica de sessão e punção única permite o tratamento imediato da veia femoral profunda, melhorando o estado clínico do paciente, garantindo afluxo adequado e possivelmente melhorando o resultado da perviedade do stent implantado a curto e longo prazo.
